# Does contact lens wear affect choroidal thickness measurements?

**DOI:** 10.1186/s40942-023-00451-2

**Published:** 2023-05-10

**Authors:** Luiz H. Lima, Lucas Z. Ribeiro, Luciana Arrais, Dante Akira, Talita F. Oliveira, Maurício Maia, Mauro S. Campos

**Affiliations:** Department of Ophthalmology, Federal University of São Paulo (UNIFESP), Rua Botucatu, 821, Vila Clementino, São Paulo, São Paulo Brazil

**To the Editor,**

The ciliary body is an inner eye structure that forms a semi-transparent ring on the anterior outer surface of the choroid. It plays a significant role in controlling accommodation, the process by which the eye adjusts optical power to provide a discernible image on an object at a varying distance. This change in focal power develops due to ciliary muscle contraction that pulls the anterior border of the choroid forward and reduces the zonular tension [1]. The purpose of this study was to measure the choroidal (CT) and retinal (RT) thicknesses in eyes of emmetropic subjects in which ocular accommodation was induced by contact lens at different refractive power.

This cross-sectional pilot study was approved by the ethics committee of Federal University of Sao Paulo, Sao Paulo, Brazil (CEP/UNIFESP n: 0819P/2021). The retinal and choroidal images of swept-source optical coherence tomography (SS-OCT) (DRI Triton, Topcon, Tokyo, Japan) were obtained in emmetropic eyes, without ocular pathology. Both the CT and RT measurements at the central macula were performed under mesopic condition before and after positive (from + 4.0 to + 4.5 D) and negative (from − 7.5 to − 11.5 D) soft contact lens wearing (Acuvue Oasys 1-day use, Johnson & Johnson Vision, Jacksonville, USA), and determined using the intrinsic automated layer segmentation software built into the SS-OCT system (DRI Triton, Topcon, Tokyo, Japan). The resulting data files consisted of the average (mean) choroidal and retina thicknesses over areas corresponding to the Early Treatment Diabetic Retinopathy Study (ETDRS) regions. The ETDRS grid was used as an overlay on the 3D macula scans. Three repeated 7 × 7 mm 3D macula scans centred at the fovea using the eye tracking were performed. Two examiners (L.A. and D.A.) who were blinded to the subjects’ clinical data scanned all participants. The results described as subfoveal CT and foveal RT considered the mean of measurements from the center (foveal area) of ETDRS grid.

An interval of 30 min between the contact lens wearing and OCT was settled to ensure ocular accommodation. CT was defined as the thickness from the outer border of the retinal pigment epithelium to the inner scleral border, and RT was defined as the thickness of the retina between the internal limiting membrane and Bruch’s membrane [2] (Fig. 1). Axial length measurements using optical biometry (IOL Master 500, Carl Zeiss, Jena, Germany) were performed in all study eyes at baseline (before the contact lens wear). Statistical analysis using the SPSS 20.0 and STATA 12 softwares was performed to evaluate the reliability of CT and RT measurements and the effect of use and type of contact lens on CT and RT at each location. The reproducibility of the CT measurements was analyzed using the Bland–Altman graph and intraclass correlation coefficient (ICC). Additional statistical tests (multiple comparisons with Bonferroni correction after linear mixed model estimation) were performed to verify difference in measurements before and after positive or negative contact lens wearing. The significance level of 5% was considered for all statistical tests.

**Fig. 1:**
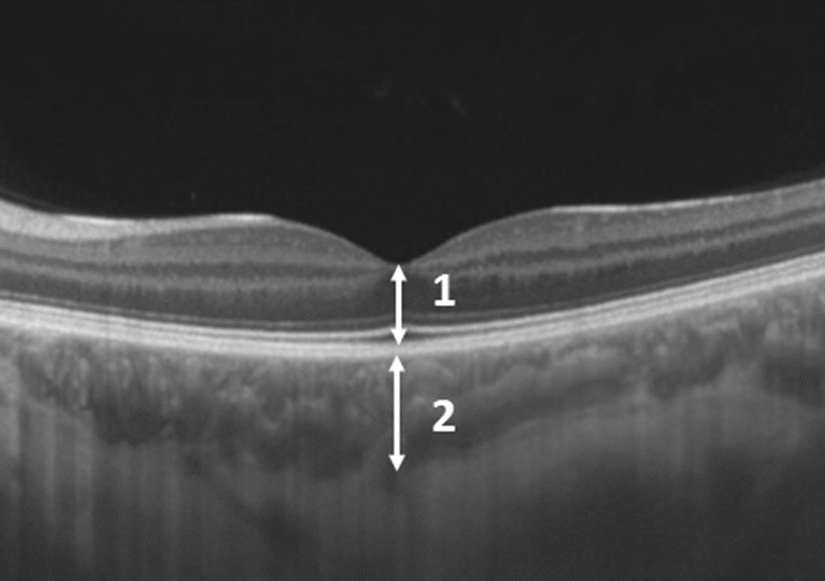
Choroidal (CT) and retinal (RT) thickness measurements. The features measured at the fovea included: (1) the CT (from the outer border of the retinal pigment epithelium to the inner scleral border), and (2) the RT (from the internal limiting membrane to the Bruch’s membrane)

The study cohort was composed of 35 eyes of 18 subjects who had a mean age of 24.0 years (standard deviation ± 3.1 years; range 19–29 years), and 10 subjects (55.6%) were female. The mean axial length measurements was 23.74 mm (range 22.34–25.11 mm). Less than 10% of the pairs of CT measurements were outside the limits of reproducibility, except for the first two CT measurements before positive contact lens wearing (11.4%). Greater differences were observed between the CT measurements with values above 340 μm. The ICC between the three CT measurements ranged from 0.972 to 0.989, indicating an excellent reproducibility (Table 1). The mean subfoveal CT was 320.9 μm (standard deviation ± 55.9 μm) and 315.4 μm (standard deviation ± 53.8 μm) before and after positive contact lens wearing, respectively. In regards to the negative contact lens, mean subfoveal CT was 320.1 μm (standard deviation ± 52.3 μm) and 316.4 μm (standard deviation ± 50.6 μm) before and after contact lens wearing, respectively. The variations of the CT averages between the different times (before and after contact lens wear) of evaluation by type (positive or negative) of contact lens were not statistically different (*p* = 0.343) (Table 2). There was no statistically significant difference between before and after positive (*p* = 0.203) or negative (*p* = 0.053) contact lens wearing.

**Table 1:** Intraclass correlation of choroidal tickness measurements by time of assessment and type of contact lens

	Interclass correlation	Confidence interval of 95%	Number of eyes	*p*
Negative contact lens				
Pre	0.972	0.952–0.985	35	< 0.001
Pos	0.981	0.967–0.990	35	< 0.001
Positive contact lens				
Pre	0.989	0.980–0.994	35	< 0.001
Pos	0.973	0.954–0.985	35	< 0.001

**Table 2:** Mean and standard deviation of choroid thickness measurements by evaluation times and type of contact lens

	Pre	Pos	Pos–pre	*p*		
	Mean (SD)	Mean (SD)	Mean (SD)	Type of contact lens	Time	Time × type of contact lens
Contact lens				0.636	0.085	0.343
Negative	320.9 ± 55.9	315.4 ± 53.8	− 5.5 ± 16.4			
Positive	320.1 ± 52.3	316.4 ± 50.6	− 3.7 ± 13.6			
	1.000	1.000	0.343			

In the subgroup of RT measurement, composed of 22 eyes of 11 subjects from the CT cohort, the ICC between the RT measurements ranged from 0.951 (pre negative lens) to 0.877 (post negative lens), indicating an excellent reproducibility. The mean foveal RT was 231.6 μm (standard deviation ± 24.3 μm) and 231.6 μm (standard deviation ± 22.2 μm) before and after positive contact lens wearing, respectively. In regards to the negative contact lens, mean subfoveal RT was 231.1 μm (standard deviation ± 23.1 μm) and 228.1 μm (standard deviation ± 25.0 μm) before and after contact lens wearing, respectively (Table 3). The variations of the RT averages between the moments (before and after contact lens wear) of evaluation by type (positive or negative) of contact lens were not statistically different (*p* = 0.148) (Table 4). There was no statistically significant difference between before and after positive (*p* = 0.276) and in the positive lens (*p* = 1.000) contact lens wearing.

**Table 3:** Intraclass correlation of retinal thickness measurements by time of assessment and type of contact lens

	Intraclass correlation	Confidence interval of 95%	Number of eyes	*p*
Negative contact lens				
Pre	0.951	0.905–0.978	22	< 0.001
Pos	0.877	0.770–0.942	22	< 0.001
Positive contact lens				
Pre	0.933	0.870–0.969	22	< 0.001
Pos	0.914	0.832–0.962	22	< 0.001

**Table 4:** Mean and standard deviation of retinal thickness measurements by time of assessment and type of contact lens

	Pre	Pos	Pos–pre	*p*		
	Mean (SD)	Mean (SD)	Mean (SD)	Type of contact lens	Time	Time × type of contact lens
Contact lens				0.168	0.138	0.148
Negative	231.1 ± 23.1	228.1 ± 25.0	− 3.0 ± 7.3			
Positive	231.6 ± 24.3	231.6 ± 22.2	− 0.1 ± 8.0			
	0.335	0.525	0.148			

The positive and negative contact lenses were chosen to induce relaxation of accommodation and the maximum power of eye accommodation, respectively, in the study subjects. With the purpose to decrease different accommodation capacities, young (mean age of 24.0 years) and emmetropic subjects represented an inclusion criteria of this study. In addition, we tested the same eye with a negative or positive contact lens, and this represented another method to reduce the possible effect of accommodative capacity.

In the present study, contact lens wear did not significantly change the OCT measurements for CT and RT in emmetropic eyes. Therefore, precise CT and RT measurements could be obtained in contact lens wearers, without the need to remove them before the OCT imaging. The interpretation of the results of the current study should be done with prudence since the results for CT and RT measurements may differ between actual hyperopic and myopic eyes, and before and after wearing in eyes that usually wear contact lenses. Also, refractive errors can influence ocular accommodation. Our study does have some limitations, such as the small sample size, and the lack of quantitative determination of the eye`s focus depth and accommodation amplitude.

## Data Availability

The datasets used and/or analysed during the current study are available from the corresponding author on reasonable request.
